# Depression and anxiety before and after bariatric surgery: a longitudinal cohort study at King Abdulaziz University Hospital, Jeddah, Saudi Arabia

**DOI:** 10.3389/fpsyt.2026.1828215

**Published:** 2026-06-16

**Authors:** Naseem Abdulmohi Alhujaili, Salhah Saleh Alsulami, Wesam Ayed Almutairi, Eman Al-khalawi, Alaa Sabbahi, Abdulaziz Abdullah Alghamdi, Murad Aljiffry

**Affiliations:** 1 Department of Medicine, Division of Psychiatry, Faculty of Medicine, King Abdulaziz University, Rabigh, Saudi Arabia; 2 Department of Medicine, Faculty of Medicine, King Abdulaziz University, Rabigh, Saudi Arabia; 3 Department of Medicine, King Abdulaziz University Hospital, King Abdulaziz University, Jeddah, Saudi Arabia; 4 Department of Family and Community Medicine, Faculty of Medicine, King Abdulaziz University, Rabigh, Saudi Arabia; 5 Department of Anesthesia and Critical Care, Faculty of Medicine, King Abdulaziz University, Jeddah, Saudi Arabia; 6 Department of Surgery, Faculty of Medicine, King Abdulaziz University, Jeddah, Saudi Arabia

**Keywords:** anxiety, bariatric surgery, depression, GAD-7, longitudinal, PHQ-9, obesity

## Abstract

**Background:**

Bariatric surgery is an effective long-term treatment for severe obesity. Psychological outcomes after surgery are clinically important.

**Methods:**

This prospective, non-interventional, longitudinal cohort enrolled adults scheduled for bariatric surgery at King Abdulaziz University Hospital (Jeddah, Saudi Arabia). The participants completed two online questionnaires: one pre-and one post-surgery (planned at approximately 6 months). Depression and anxiety symptoms were assessed using the Patient Health Questionnaire-9 (PHQ-9) and Generalized Anxiety Disorder-7 (GAD-7). Positive screening was defined as PHQ-9 ≥10 and GAD-7 ≥10. Weight was self-reported using the questionnaires. Post-surgical complications were self-reported in the post-surgery questionnaire as ‘yes/no’ items. Pre- and post-surgery scores were compared using the paired t-test. Sensitivity analysis was assessed using the Wilcoxon signed-rank test. Proportions that screened positive were compared using the Z-test. Univariate linear regression was used to assess the association between symptom changes and demographic, clinical, and surgical factors. Analyses were conducted using Stata/AC v18.

**Results:**

Twenty-seven participants completed the pre-surgery questionnaire, and 23 completed the post-surgery questionnaire. The matched analyses included 23 participants. The mean (SD) age was 33.6 (10.4) years, and 11 (47.8%) were female. Although follow-up was planned at approximately 6 months, the completion time varied (mean 359.5 [273.5] days; median 224; range 84–906). The mean (SD) GAD-7 decreased from 6.0 (5.9) to 5.2 (5.5) (p=0.565), while the mean (SD) PHQ-9 decreased from 7.5 (7.3) to 5.9 (6.4) (p=0.430). The proportion screening positive decreased from 26.1% to 17.4% for GAD-7 (p=0.475), and from 26.1% to 13.0% for PHQ-9 (p=0.265). In the univariate models, post-surgical complications were associated with worse anxiety and depression trajectories, while prior anxiety diagnosis was associated with greater improvement in anxiety symptoms.

**Conclusion:**

In this cohort, the average depression and anxiety scores declined after bariatric surgery, although the overall changes were not statistically significant. Self-reported post-surgical complications were consistently associated with worse psychological outcomes. Our findings support the need for integrated psychiatric follow-ups during the bariatric surgical evaluations, before surgery and after surgery, particularly in patients with reported complications.

## Introduction

Obesity is a global public health problem that has become increasingly problematic in recent decades (1). Obesity is the leading cause of morbidity and mortality, with serious physical and mental implications (2). Obesity is defined as excessive accumulation or improper distribution of body fat (BF), which, negatively impacts people’s health (3). Body mass index (BMI; kg/m2) is widely used to classify a person’s body weight into underweight, normal weight, overweight, or obese (4). According to “the Expert Panel on the Identification, Evaluation, and Treatment of Overweight and Obesity in Adults, “ obesity is defined as a BMI of ≥30 kg/m2 (5). Obese people are at a higher risk of several psychiatric disorders (e.g., depressive disorder) and non-psychiatric diseases (e.g., diabetes mellitus and cardiovascular disease) than non-obese people (6).

In 2020, the prevalence of obesity in Saudi Arabia was 24.7% (7). According to a World Health Organization (WHO) report, the obesity prevalence among Gulf countries is similar, with 19-36% in males and 32-48% in women (8). Recent obesity reports revealed that more than 20% of obese Saudi adults have a BMI of more than 35. Consequently, obese patients are encouraged to reduce their weight in various ways. Weight loss responses vary depending on patient compliance and a variety of other factors (9, 10).

According to Swedish patients with obesity, bariatric surgery is the most successful long-term treatment for morbid obesity (11). In Saudi Arabia, more than 20000 bariatric surgeries are performed annually, and over 24 surgeries occur weekly at King Khaled University in Riyadh (9). Various laparoscopic bariatric surgeries, including laparoscopic sleeve gastrectomy (LSG), Roux-en-Y gastric bypass surgery (RYGB), and adjustable gastric banding (AGB), have become more prevalent as successful obesity treatment options (12, 13).

Obese people who seek treatment for obesity have higher rates of depression than those who do not seek such intense weight control measures (14). Consequently, it is not surprising that many patients’ seeking surgical intervention for obesity are battling depression (15). While bariatric surgery is generally good for obese patients with depression, other weight loss interventions have also been demonstrated to reduce stress and improve quality of life (16, 17). Importantly, there is a subset of people who may become more depressed after the surgical procedure. Ivezaj et al. found that approximately 15% of patients will experience worsening moods between 6 and 12 months postoperatively (18). The worsening of mental symptoms in this group of patients is likely multifactorial. The reasons may include inadequate weight loss, unrealistic expectations, or other life stresses that need to be appropriately monitored after surgery. Importantly there appears to be a link between suicide rates and bariatric surgery (19). Postoperative depression has also been associated with poor overall weight loss. Therefore, determining the link between bariatric surgery and depression is crucial for long-term success of weight loss (20).

This study aimed to assess the prevalence of depression and anxiety pre- and post-bariatric surgery and to explore factors associated with post-surgical depression and anxiety, such as age, sex, amount of weight loss, and medical comorbidities.

To our knowledge, studies that examined the prevalence of depression and anxiety pre- and post-bariatric surgery in Saudi Arabia are scarce. This study aimed to fill this knowledge gap in the literature. Moreover, the study aimed to enhance the awareness among healthcare providers of the link between bariatric surgery and depression/anxiety, which is crucial to the success of the procedure. Furthermore, our findings may contribute to a better analysis of whether more psychological support services are needed for patients before and/or after bariatric surgery.

## Methods

### Participants and data collection

This study was approved by Unit of Biomedical Ethics at King Abdulaziz University (Reference number: 165-22). Prior to participation, verbal consent was obtained by phone, and electronic consent was obtained through an online questionnaire. Approval date: April 6, 2022.

This prospective, non-interventional, longitudinal cohort study was conducted in the surgical clinics of King Abdulaziz University Hospital (KAUH) in Jeddah, Saudi Arabia. Adults (≥18 years) scheduled for bariatric surgery including (sleeve gastrectomy, and Roux-en-Y gastric bypass).

The exclusion criteria were age<18 years, inability to provide informed consent, and those with obesity but did not undergo bariatric surgery.

The sample size calculated as follows: = ${\left(1.28+1.96\right)}^{2}(\sigma 1^{2}+\sigma 0^{2})÷{\left(\mu 1-\mu 0\right)}^{2}$


$$={\left(1.28+1.96\right)}^{2}\left(5^{2}+5^{2}\right)÷{\left(5\right)}^{2}=21$$

We added 20% to account for potential loss to follow-up, resulting in a total of 25 participants required.

The list of patients scheduled for bariatric surgery was obtained from the Surgical Department at KAUH. The research team contacted patients who fulfilled the inclusion criteria. As part of the informed consent process, the purpose of the study and the right of the participants to withdraw at any time without any impact on their treatment plan were explained.

After obtaining verbal consent, a link to the preoperative questionnaire was sent to the participants two months before their scheduled bariatric surgery. Six months postoperatively, another link was sent to the participants, inviting them to complete the postoperative questionnaire. In total, the participants completed two online questionnaires: one pre-surgery, which was sent approximately two months before their bariatric surgery, and one post-surgery, which was sent approximately 6 months after the procedure.

### Measurements

Both pre- and post-surgery questionnaires included consent, [participant’s study number, demographic data, medical history, past psychiatric history, the Patient Health Questionnaire (PHQ)-9 for screening depression (Validated in Arabic) (21), and the Generalized Anxiety Disorder 7-item (GAD-7) for screening anxiety (Validated in Arabic) (21).

#### PHQ-9

Depressive symptoms were assessed using the Patient Health Questionnaire-9 (PHQ-9), a validated 9-item self-report scale. Each item is scored from 0 to 3, with total scores ranging from 0 to 27; higher scores indicate greater depressive symptom severity.

#### GAD-7

Anxiety symptoms were assessed using the Generalized Anxiety Disorder-7 (GAD-7), a validated 7-item self-report scale. Each item is scored from 0 to 3, with total scores ranging from 0 to 21; higher scores indicate greater anxiety severity.

Positive screening thresholds for depression and anxiety were defined as PHQ-9 ≥10 GAD-7 ≥10, respectively.

Weight was self-reported by the participants in both, the pre- and post-surgery questionnaires. Post-surgical complications were self-reported in the post-surgery questionnaire as ‘**yes/no’** items.

### Statistical analysis

Twenty-seven patients participated in the pre-surgery survey. Twenty-three responded to the post-surgery survey. Participants in both surveys were included in the analysis (n=23). Continuous variables are described using means and standard deviations, or medians and ranges if the data were skewed. Categorical variables are described using frequencies and percentages. The PHQ-9 and GAD-7 scores were calculated for each participant, and the proportion of those who scored as likely to suffer from depression or anxiety was calculated according to the tool’s key.

A paired t-test was used to compare pre- and post-surgery GAD-7 and PHQ-9 scores. A Wilcoxon signed-rank test was also used, given the small sample size and potential non-normality of the score differences. The Z-test was used to examine the difference in the proportion of patients with overall positive screening test results before and after surgery.

Linear regression was used to examine the association between various demographic, health, and procedure-related variables and changes in the PHQ-9 and GAD-7 scores. The Shapiro-Wilk test was used to test for the normality of residuals, which deviated significantly from normality (p<0.05), and robust standard errors were used to reduce sensitivity to non-constant variance in the residuals. Analyses were performed using Stata AC version 18.

## Results

The mean age of the participants was 33.6 years (±10.4), and just under one half were female (47.8%). The majority had a university education (78.3%). Among the participants, 65.2% had at least one chronic condition, the most common being osteoarthritis, followed by hypertension, diabetes, and fatty liver. Approximately one quarter had a pre-existing diagnosis of psychiatric conditions. Approximately three-quarters of the patients underwent gastric sleeve surgery, while the remaining patients underwent gastric bypass surgery. Six participants (25%) reported post-surgical complications, the most common was gastroesophageal reflux disease GERD (16.7%), followed by postoperative hematoma and pulmonary embolism (4.2%) for each. Patients were followed up for almost one year on average (± 273.5 days) (**Table 1**).

**Table 1:** Participant characteristics (n=23).

Charactertics		Mean	SD
Age	(Years)	33.6	10.4
Hight	(cm)	164.6	9.8
Weight before surgery	Mean (Kg)	122.5	30.1
	median, range	113	85-198
Weight after surgery	Mean (Kg)	83.5	20.5
	median, range	83	43-120
Weight loss	Mean (Kg)	39	20.5
	median, range	34	0-88
Follow-up time	Mean (days)	359.5	273.5
	median (days)	224	84-906
		Frequency	%
Sex	Female	11	47.8
	Male	12	52.2
Marital status	Married	10	43.5
	Single	10	43.5
	Divorced	2	8.7
	Widowed	1	4.4
Education	Primary	1	4.35
	Secondary	4	17.39
	University	16	69.57
	Higher education	2	8.70
Occupation	Student	4	17.39
	Freelance	3	13.04
	Unemployed	4	17.39
	Employed	12	52.17
Chronic medical conditions	Osteoarthritis	7	30.4
	Difficulty conceiving	2	8.7
	Fatty liver	3	13
	Hypertension	4	17.4
	Gallstones	1	4.4
	Diabetes	3	13
	Polycystic ovarian syndrome	1	4.4
	Hyperlipidemia	1	4.4
	Hyperthyroidism	1	4.4
Number of chronic medical conditions	None	8	34.78
	One	9	39.13
	Two	4	17.39
	Three	2	8.7
Psychiatric condition diagnosis	Anxiety	1	4.4
	Depression	2	8.7
	Eating disorder	1	8.7
	Sleep disorder	2	4.4
Surgery type	sleeve gastrectomy	17	73.91
	Roux-en-Y gastric bypass	6	26.09
Post-surgical complications	Yes	6	25%

The mean and median scores for both the GAD-7 and PHQ-9 were lower after surgery, but the differences were not statistically significant, and there was no change in the proportion of those who had a positive screening test using these two tools (**Table 2**).

**Table 2:** Difference in anxiety and depression scores before and after bariatric surgery.

	Before surgery		After surgery		Difference	P-value
Score	Mean	SD	Mean	SD		
GAD-7 score	6.0	5.9	5.2	5.5	0.78	0.565^a^
PHQ-9 score	7.5	7.3	5.9	6.4	1.61	0.430^a^
	Median	Range	Median	Range		
GAD-7 score	5	0-21	4	0-19		0.116^b^
PHQ-9 score	6	0-23	3	0-23		0.253^b^
	Frequency	%	Frequency	%		
Positive GAD-7	6	26.1	4	17.4	8.70	0.475^c^
Positive PHQ-9	6	26.1	3	13.0	13.0	0.265^c^

^a^ Paired t-test for equal means.

^b^ Wilcoxon signed-rank test.

^c^ Z-test for equal proportions.

None of the demographic or medical characteristics were significantly associated with a change in the GAD-7 or PHQ-9 scores from before to after surgery, except for a previous diagnosis of an anxiety disorder. Participants with a pre-existing anxiety disorder demonstrated a 6.3-point decrease in the GAD-7 score (p<0.001). Additionally, post-surgical complications were associated with a 6.9 point increase in the GAD-7 score (p=0.03) (**Table 3**) and a 12-point increase in the PHQ-9 score (p<0.001) (**Table 4**).

**Table 3:** The associations between different variables and the anxiety score before and after surgery (univariate).

Variable	Coefficient	Standard error	P-value	95% CI	
				Lower	Upper
Age	-0.2	0.1	0.14	-0.50	0.07
Gender	0.6	2.7	0.83	-4.98	6.16
Marital status (ref: married)					
Single	2.2	6.5	0.74	-11.38	15.78
Married	-3.8	6.5	0.57	-17.38	9.78
Divorced	-1.0	7.6	0.90	-16.86	14.86
Occupation (ref: student)					
Freelance	-5.7	5.2	0.29	-16.63	5.30
Unemployed	-7.5	5.4	0.18	-18.83	3.83
Employed	-7.2	5.4	0.20	-18.42	4.08
Previous diagnosis of anxiety	-6.5	1.4	<0.001	-9.41	-3.59
Number of previously diagnosed psychiatric conditions	-0.9	2.7	0.76	-6.57	4.84
Number of previously diagnosed medical conditions	-2.3	1.5	0.15	-5.35	0.85
Weight loss (Kg)	0.0	0.0	0.75	-0.09	0.06
Surgery type (bypass compared to sleeve)	-2.8	3.1	0.38	-9.26	3.71
Post-surgical complications (yes)	6.9	3.0	0.03	0.71	13.05
Number of medical conditions after surgery	-4.4	3.0	0.15	-10.62	1.78

**Table 4:** The associations between different variables and the depression score before and after surgery (univariate).

Variable	Coefficient	Standard error	P-value	95% CI	
				Upper	Lower
Age	0.24	0.17	0.17	-0.11	0.60
Gender	-2.67	3.60	0.47	-10.16	4.83
Marital status (ref: married)					
Single	1.40	8.90	0.88	-17.23	20.03
Married	7.60	8.90	0.40	-11.03	26.23
Divorced	8.00	10.40	0.45	-13.76	29.76
Occupation (ref: student)					
Freelance	4.17	6.66	0.54	-9.77	18.11
Unemployed	8.50	6.17	0.18	-4.40	21.40
Employed	5.92	5.03	0.25	-4.62	16.45
Previous diagnosis of depression	0.43	6.47	0.95	-13.03	13.88
Number of previously diagnosed medical conditions	3.20	4.76	0.51	-6.70	13.09
Number of previously diagnosed psychiatric conditions	3.20	1.65	0.07	-0.23	6.63
Weight loss (Kg)	-0.03	0.09	0.70	-0.21	0.15
Surgery type (bypass compared to sleeve)	0.75	4.15	0.86	-7.87	9.38
Post-surgical complications (yes)	-12.02	3.56	<0.001	-19.42	-4.62
Number of medical conditions after surgery	6.31	3.72	0.10	-1.41	14.04

Overall, anxiety and depression scores decreased after surgery. However, those who experienced post-surgical complications had higher anxiety and depression scores after surgery on average.).

## Discussion

This study examined the psychological outcomes of bariatric surgery, focusing on depression and anxiety before and after the procedure, as well as the influence of various demographic and medical factors. Bariatric surgery can lead to long-term improvements in depression and anxiety. In our study, the mean and median scores of the GAD-7 and PHQ-9 decreased following surgery. However, the changes in anxiety and depression score were not statistically significant in the overall sample. This is aligned with international data from systematic review of 14 studies that followed patients over time, 13 studies (93%) found clear and meaningful decreases in depressive symptoms for up to three years after surgery. Anxiety symptoms also reduced in patients who were followed for two years or more after surgery (22). This is supported by previous studies that found bariatric surgery is helpful for obese patients with depression (16, 17). This finding contrasts with other studies in which patients experienced worsening depressive symptoms during the short- and intermediate-term postoperative period (18). Obesity is often associated with reduced This improvement in depression and anxiety may be attributed to factors such as increased self-esteem, self-esteem, which can positively impact psychological well-being and quality of life.

Most bothersome postoperative complications were GERD (16.7%). Participants who experienced post-surgical complications showed a significant increase in anxiety and depression scores. This pattern suggests that complications following bariatric surgery may negatively influence psychological well-being, potentially through prolonged recovery, functional limitations, or concerns about surgical success. This aligned with prior findings suggesting that complications, such as gastroesophageal reflux, infection, or pain, can contribute to heightened psychological distress post-operatively (23).

Patients with a pre-existing anxiety diagnosis showed the most pronounced improvement in the GAD-7 postoperatively (mean score reduction of 6.3, p<0.001). Although direct comparison is not appropriate due to the lack of subgroup analysis by surgical type. This is consistent with previous studies which reported that bariatric surgery reduced the prevalence and severity of various psychiatric conditions (24, 25). As following bariatric surgery at short and intermediate postoperative course anxiety and depression significantly reduced (25). This can be explained by improvement in obesity-related complications, which may contribute to reduced anxiety levels. Additionally, the rapid improvement in anxiety may be related to the resolution of surgery-related stress. However, further studies are needed to draw more robust conclusions.

Several studies have demonstrated a clear association between depression and elevated levels of inflammatory biomarkers, including interleukin-6 (IL-6), tumor necrosis factor-alpha (TNF-α), and C-reactive protein (CRP). A meta-analysis by Strawbridge et al. (2015) confirmed that an increase in inflammatory markers is commonly observed in individuals with depression and may influence treatment response (26). In a recent longitudinal study, Jensen et al. (2025) evaluated cytokine levels in 68 severely obese patients before and after bariatric surgery. Their findings revealed a significant reduction in inflammatory cytokine levels 12 months after surgery, regardless of the surgical technique used (27). Similar results were reported in the 2019 systematic review and meta-analysis by Askarpour et al., which assessed the impact of bariatric surgery on IL-6, TNF-α, and CRP levels. The analysis showed a statistically significant decrease in all three inflammatory markers after surgery. These findings support the anti-inflammatory benefits of weight loss surgery (28). These reductions in inflammatory biomarkers post-bariatric surgery play a role in improving metabolic health and in alleviating depression and anxiety.

Interestingly, variables, such as age, sex, weight loss, and type of surgery (sleeve vs. bypass), were not significantly associated with changes in anxiety or depression. Although the absence of statistically significant overall improvements may initially appear disappointing, the direction of the scores indicated a trend toward less psychological distress, which is promising and clinically relevant. Our results are consistent with the findings of other studies, which found that bariatric surgery often leads to psychiatric improvements (14, 29).

Taken together, the current findings highlight the need for integrated psychological care in bariatric surgery programs, particularly for patients with prior psychiatric diagnoses and those who experience complications. Ongoing mental health support, patient education, and early interventions for postoperative issues may mitigate the risk of worsening psychiatric symptoms in this vulnerable subgroup.

This study has some limitations, including potential recall bias, and using screening tools such as PHQ-9 and GAD-7 rather than clinical diagnoses. The analyses were conducted using pre–post comparisons and univariate regression models; however, given the limited sample size, the regression findings should be interpreted with caution. Additionally, self-reported weight and complications may introduce reporting bias. In addition, baseline psychiatric conditions are an important confounding factor that may not have been fully adjusted for in the multivariable regression models. Moreover, direct comparison with other bariatric surgery studies should be made cautiously, as the surgical type in our cohort was not available for subgroup analysis. Finally, variability in follow-up duration, despite a minimum of 6 months, may introduce heterogeneity and act as a potential confounding factor in the assessment of depression and anxiety outcomes. Future studies with sample sizes, multi-center cohort designs, longitudinal postoperative assessments, and detailed qualitative data to better understand the patient perspectives are warranted. Despite this limitation, a key strength of our study is its longitudinal design, with patients followed over time and assessed at two distinct points, enabling direct pre- and post-bariatric surgery comparisons.

## Conclusion

In this longitudinal study, preliminary findings suggest a reduction in mean depression and anxiety scores following bariatric surgery; however, these changes were not statistically significant. Self-reported post-surgical complications were associated with poorer psychological outcomes. These results underscore the importance of integrating psychological assessment and support into bariatric care pathways, particularly for patients experiencing post-operative complications.

## Data Availability

The original contributions presented in the study are included in the article/supplementary material. Further inquiries can be directed to the corresponding author.
